# A Theoretical Treatment of THz Resonances in Semiconductor GaAs p–n Junctions

**DOI:** 10.3390/ma12152412

**Published:** 2019-07-29

**Authors:** Mohsen Janipour, I. Burc Misirlioglu, Kursat Sendur

**Affiliations:** Faculty of Engineering and Natural Science, Sabanci University, 34956 Istanbul, Turkey

**Keywords:** semiconductor plasmonics, semiconductor heterojunctions, plasmonic waveguide, p–n junction

## Abstract

Semiconductor heterostructures are suitable for the design and fabrication of terahertz (THz) plasmonic devices, due to their matching carrier densities. The classical dispersion relations in the current literature are derived for metal plasmonic materials, such as gold and silver, for which a homogeneous dielectric function is valid. Penetration of the electric fields into semiconductors induces locally varying charge densities and a spatially varying dielectric function is expected. While such an occurrence renders tunable THz plasmonics a possibility, it is crucial to understand the conditions under which propagating resonant conditions for the carriers occur, upon incidence of an electromagnetic radiation. In this manuscript, we derive a dispersion relation for a p–n heterojunction and apply the methodology to a GaAs p–n junction, a material of interest for optoelectronic devices. Considering symmetrically doped p- and n-type regions with equal width, the effect of certain parameters (such as doping and voltage bias) on the dispersion curve of the p–n heterojunction were investigated. Keeping in sight the different effective masses and mobilities of the carriers, we were able to obtain the conditions that yield identical dielectric functions for the p- and n-regions. Our results indicated that the p–n GaAs system can sustain propagating resonances and can be used as a layered plasmonic waveguide. The conditions under which this is feasible fall in the frequency region between the transverse optical phonon resonance of GaAs and the traditional cut-off frequency of the diode waveguide. In addition, our results indicated when the excitation was slightly above the phonon resonance frequency, the plasmon propagation attained low-loss characteristics. We also showed that the existence or nonexistence of the depletion zone between the p- and n- interfaces allowed certain plasmon modes to propagate, while others decayed rapidly, pointing out the possibility for a design of selective filters.

## 1. Introduction

The conductivity response of a junction formed between a semiconductor (SC) and a metal or a dielectric, upon application of a voltage bias, has been at the core of the semiconductor-based solid state devices that led to the electronic revolution. The electronic characteristics of such a junction can be engineered via the choice of the materials and the doping on the SC side, to achieve a desired response. Since the first appearance of semiconductor heterostructures, the sizes of devices have been considerably reduced to submicron scales, owing to the advances in fabrication capabilities. In integrated circuits (IC), the main action of a semiconductor heterojunction is often whether to allow a current to pass or not, depending on the applied bias voltage and its sign. This is determined by the width of the depletion zone in a Schottky-type or p–n-type junction. The former occurs upon contact of a metal with a semiconductor and the latter forms between dissimilarly doped semiconductors.

Apart from their conductivity related applications, the idea to use semiconductor heterojunctions for optical manipulation emerged in the 1960s, when a number of works analyzed electromagnetic wave transmission along a p–n junction, at the millimeter scale, and revealed some interesting optical physics in such systems [1,2]. Most notably, during the past decade, studies on the unique role of surface plasmon polaritons (SPPs) that allow propagation of light through subwavelength nanostructures has attained great interest in developing nano-photonic integrated circuits for a number of purposes [3,4]. The concept of SPPs coupled to specific excitation conditions has led to the development of various kinds of waveguides in the visible light regime [5,6,7,8]. Among these, due to their capability of photonic confinement, noble metallic based multilayer metal-insulator-metal (MIM) layers in the visible frequency regime has been widely studied by several researchers [9,10,11]. 

The interaction of light with the electrons of noble metals at metal-dielectric interfaces of the MIM waveguides can result in a much better SPP confinement due to the electromagnetic coupling of the localized free electron oscillations to the incoming excitation [10]. In addition to the noble metals, D. Y. Fedyanin et al. [12], A.V. Krasavin et al. [13], R. Zektzer et al. [14], and O. Lotan et al. [15] have shown that Cu, Si, and Al-based structures can also provide SPP guiding channels in the visible and infrared (IR) regime. To achieve such plasmonic effects, other semiconductors like GaAs can also be considered in which free carriers of negative or positive signs with appropriate effective masses can populate either the conduction band or the valence band, respectively, via appropriate doping. GaAs has also been the choice for applications, including manufacturing of microwave integrated circuits [16], infrared light emitting diodes [17], laser diodes [18], and solar cells [19]. In addition, plasmonic effects in GaAs can enable hybrid electro-optic/photonic integrated devices with high performance, easy-fabrication, and tunable properties, with substantially high propagation length in comparison with the noble metals [20,21,22,23,24]. Consequently, applying the idea of doping to the multi-layered semiconductor heterostructure configuration, several applications like plasmonic optical modulators, waveguides, and meta-materials have been presumed for these novel photo-plasmonic devices in the IR and THz frequencies [25,26,27,28,29,30]. Luther et al. [31] and Williams et al. [32] have experimentally shown that similar tunable localized surface plasmon resonances (LSPR) can be achieved in doped semiconductor quantum dot structures for wave-guiding in the THz and IR regime [24,25,26,27,28,29,30,31,32,33]. The latter has also been shown for layered metal-dielectric-semiconductor and Schottky junctions, which can enable nanoscale SPP amplifiers using an electrical pump injected to the configuration [34,35,36,37]. Moreover, Fan et al. [38] showed that the electrically driven GaAs nanowire light sources can be coupled to plasmonic nano-strip waveguides. It has also been numerically shown that by tuning the positive voltage bias of a highly p–n-doped diode, a Y junction optical switch can be obtained through the propagation of SPPs [39].

As semiconductors allow electric field penetration and possess carrier densities that can allow resonances, at least in theory, in the THz frequencies, we explore the characteristics of a p–n-heterojunction for plasmonics. We demonstrate that, the existence/absence of the depletion zone at a p–n junction can act as a plasmonic filter for frequencies in the THz regime. The classical dispersion relations in the literature are already derived for metals, such as gold and silver, interfacing a dielectric for which a homogeneous dielectric function is valid. However, for semiconductor materials under an applied voltage, such as the p–n heterojunction, the dielectric constant varies as a function of coordinates resulting from the inhomogeneous electric field penetration. In this manuscript, we first derived a dispersion relation for the p–n heterojunction. Using these dispersion relations, we theoretically and numerically investigated the plasmonic wave-guiding mechanism of a GaAs-based p–n junction, at different doping densities. We carried out the analysis under various applied bias values. For the GaAs system, we showed that when the excitation is slightly above the phonon resonance frequencies, the plasmon propagation attains a low-loss characteristic, which is highly attractive for plasmon propagation applications. We also showed that the existence or nonexistence of the depletion zone between the p- and n- interfaces, controlled by applied bias, allows selective modes to propagate while others decay rapidly. One can design submicron devices around the concepts presented herein with plasmon-driven frequency selectivity in the optical regime.

## 2. Material Properties

GaAs is a III-V direct bandgap semiconductor with a zinc-blende crystal configuration [40]. Varga previously showed for GaAs that in the long-wavelength region, the lattice vibrations and the conduction electrons have a combined contribution to its dielectric function [41]. Furthermore, several studies have investigated the interaction of bulk plasmons with optical phonons in the THz regime for the doped GaAs medium [24,42,43,44]. Although in the p-doped GaAs, the hole mobility is very low (i.e., $\mu_{p}=400\;{\mathrm{cm}}^{2}\cdot V^{-1}\cdot s^{-1}$), the electron mobility in an n-doped GaAs medium is comparable (i.e., $\mu_{n}\leq 8500\;{\mathrm{cm}}^{2}\cdot V^{-1}\cdot s^{-1}$) with those reported for graphene films (i.e., $\mu_{n}\approx 15000\;{\mathrm{cm}}^{2}\cdot V^{-1}\cdot s^{-1}$), which can in principle allow the use of the GaAs medium as an optical waveguide in certain frequencies and doping values. GaAs system is attractive for the levels of doping that can be reached in this system without sacrificing the lattice stability, as well as the high mobility of the carriers among semiconductors, including Si. Controlled doping combined with high carrier mobility could in principle allow THz resonances in a semiconductor and GaAs is an almost ideal platform material for this end. MIM systems, on the other hand, are more suitable for visible and IR regions of the spectrum where the carrier mobilities and relaxation times can support resonances in the relevant spectral regime. The fact that carrier density can be controlled by an external DC bias in a semiconductor lattice, provides the added functionality of resonance tunability that is otherwise absent in MIM structures.

In this section, the *m_e_* is the electron mass, $V_{bi}$ refers to the built-in potential, $\tau_{j}$ is the carrier relaxation time of the majority carriers in the relevant p- and n-doped regions, and $\gamma_{j}$ is the damping frequency of the majority carriers in the relevant p- and n-doped regions and $\tau_{j}=1/\gamma_{j}$. In general, for a bulk GaAs medium, one can represent the optical dielectric function as:

(1) $$\varepsilon_{j-GaAs}(\omega )=\varepsilon_{\infty ,GaAs}\left(1-\frac{\omega_{pj}^{2}}{\omega (\omega +i\gamma_{j})}\right)+\frac{(\varepsilon_{DC,GaAs}-\varepsilon_{\infty ,GaAs})\times \omega_{TO}^{2}}{\omega_{TO}^{2}-\omega^{2}-i\omega \Gamma },$$

where, $\varepsilon_{\infty ,GaAs}$ and $\varepsilon_{DC,GaAs}$ are the high-frequency and static dielectric constant of GaAs, *j* = *p*, *n*, $\omega_{pj}=\sqrt{N_{j}\times e^{2}/(\varepsilon_{0}\varepsilon_{\infty ,GaAs}m_{j}^{*})}$, where e is the electron charge, $N_{j}$ is the carrier concentration and $\gamma_{j}$ represent the plasma and damping frequency of the majority carriers in the relevant p- and n-doped regions, respectively. The electron and hole effective masses in Equation (1) are assumed as $m_{n}^{*}$ = 0.067 × $m_{e}$, and $m_{p}^{*}$ = $\left(\sqrt[{3}]{m_{lh}^{2}}+\sqrt[{3}]{m_{hh}^{2}}\right)$/$\left(\sqrt{m_{lh}}+\sqrt{m_{hh}}\right)$, with $m_{lh}=0.53\times m_{e}$ and $m_{hh}=0.08\times m_{e}$ as the light-hole and heavy-hole effective masses, respectively. We have also calculated the static conductivity of the bound holes and electrons in the doped GaAs using $\sigma =\sigma_{ps}+\sigma_{ns}$ where $\sigma_{js}=\pm e\times N_{j}\times \mu_{j}$ in which $\mu_{j}$ is the mobility of the hole and electron, respectively. In addition, to calculating the damping frequencies in Equation (1), the carrier relaxation time in the doped GaAs is computed using the formula $\tau_{j}=m_{j}^{*}\sigma_{ps}/N_{j}e^{2}$, so that the p- and n-doped regions and are approximately $\tau_{p}=92\times 10^{-15}$ s and $\tau_{n}=324\times 10^{-15}$ s, which are much larger than the values of gold and silver (i.e., τ=30−40 fs). In Equation (1), $\omega_{TO}$ and Γ denote the transverse optical (TO) phonon resonance and damping phonon frequency, respectively, which are considered independent of the doping densities [45,46,47] and are summarized in Table 1.

**Figure 1:**
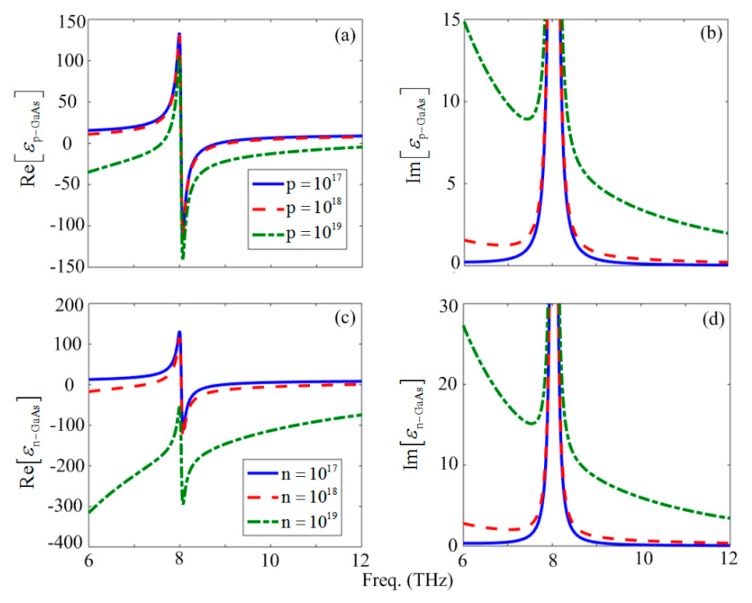
The spectral variation of the (**a**,**c**) real and (**b**,**d**) imaginary parts of the dielectric functions of the p- and n-GaAs for $N_{p,n}=10^{17}({\mathrm{cm}}^{-3})$ [solid curve], $N_{p,n}=10^{18}({\mathrm{cm}}^{-3})$ [dashed curve], and $N_{p,n}=10^{19}({\mathrm{cm}}^{-3})$ [dashed-dotted curve], respectively.

Figure 1a,c and Figure 1b,d, demonstrate the effect of p- and n-type dopants on real and imaginary parts of the dielectric function for $N_{p,n}=10^{17}({\mathrm{cm}}^{-3})$ [solid curve], $N_{p,n}=10^{18}({\mathrm{cm}}^{-3})$ [dashed curve], and $N_{p,n}=10^{19}({\mathrm{cm}}^{-3})$ [dashed-dotted curve], respectively. Please note that such doping levels have been reported for GaAs, such as in the case of carrier mobility studies [48], as well as lattice stability of GaAs [49] and device design [50]. However, such aggressive atomic doping concentrations are still challenging to achieve in practical applications, as the zinc blende GaAs has approximately 4.5 × 1022 atoms/cm^3^. In Figure 1a–d it can be seen that the n-GaAs exhibits larger negative real and positive imaginary parts of the dielectric function in comparison to the p-GaAs. This is due to the lighter carrier effective mass in the conduction band than for holes in the valence band. For a constant doping density, by increasing the frequency, a much higher negative value of the real part and greater imaginary values can be obtained. Furthermore, in Figure 1a,c and Figure 1b,d, it can be seen that although the phonon resonant frequency of the lattice is considered independent of the doping densities, the phonon-plasmon interactions are substantial for the relatively heavily doped cases. The real part of the dielectric function at frequencies before the TO phonon resonance frequency is strongly affected by the doping density that tends to have a more negative value. This property is significant in the n-doped GaAs in comparison to the p-GaAs. However, at certain frequencies it can be seen from Figure 1b,d that the imaginary part of the dielectric function in the p-GaAs is approximately half of that of the n-GaAs. These optical properties make the doped GaAs an attractive candidate for the novel plasmonic materials in the THz regime.

Keeping this behavior in mind, with the electronic features like charge distribution and band diagram of the semiconductor-metal interfaces, one can consider the layered plasmonic waveguide structures [51,52]. The plasmonic waveguide idea is centered around the concept of the gas oscillation model of free electrons in the visible regime, where under phase-matched conditions, the energy of the illuminating photons can be coupled to the free electrons of the noble metals at the metal–dielectric interface, which can overcome the diffraction limits at nanoscale [11]. This behavior is a unique feature of the noble metals at visible and near-infrared frequencies. At lower frequencies like gigahertz, terahertz, and the far infrared (FIR) regime, the optical properties of the metals are not attractive for plasmonics [24,39]. In the mid-IR regime, the optical properties of the GaAs medium can be analyzed via the Drude model, and the influence of the optical phonons is weak [53]. As we demonstrate in the following sections, an engineered p–n junction diode can provide alternative configurations owing to their inherent carrier transport characteristics at GHz and THz regimes where metals are no longer functional.

## 3. Dispersion Relation for the p–n Junction of an Inhomogeneous Dielectric Constant

**Figure 2:**
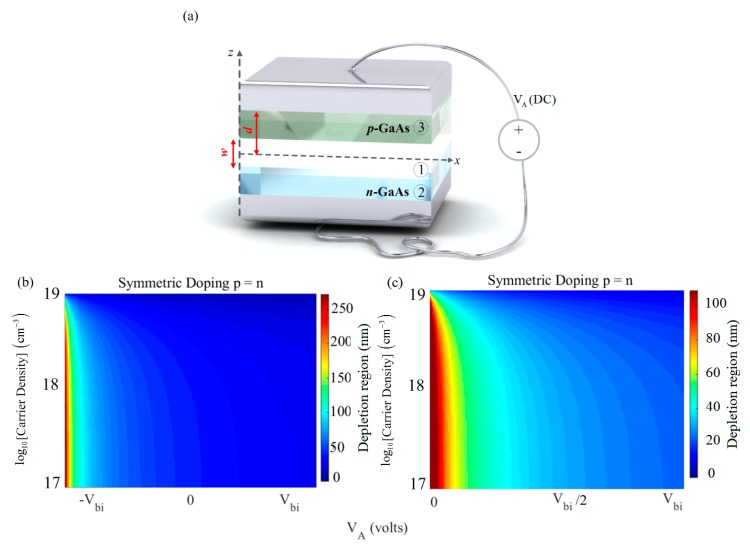
(**a**) Schematic representation of the considered structure; (**b**,**c**) variation of the depletion region width versus different carrier densities (in logarithmic scale) and applied bias voltage for the symmetric doping case $-V_{bi}\leq V_{A}\leq V_{bi}$, and $0\leq V_{A}\leq V_{bi}$, respectively.

To study the interaction of optical phonons with carriers and their resultant effect on the plasmon propagation in the GaAs p–n junction interfacing metal electrodes (Figure 2), it is first worth noting that in Figure 1a–d, the pure plasmons caused by the Drude model (before $\omega_{TO}$) were lossy. Due to this property of the plasmons, we focused on the frequency region around $\omega_{LO}$, which showed a smaller imaginary part (low-loss) of the permittivity.

Figure 2a illustrates the schematic representation of the GaAs p–n junction under the external bias condition. First, we considered the symmetrical doped p- and n-doping regions with an equal width of *d* = 500 nm and $-V_{bi}\leq V_{A}\leq V_{bi}$. For the biased p–n-diode, the width of the depletion region could be easily obtained by $w\approx \sqrt{\frac{2\varepsilon_{DC,GaAs}\varepsilon_{0}}{e}\sum_{j=p,n}\left(1/N_{j}\right)\times (V_{bi}-V_{A})}$, such that $\varepsilon_{DC,GaAs}=$ 12.9 was the static dielectric constant of GaAs [52]. Considering the negative bias voltage values [i.e., $-V_{bi}\leq V_{A}\leq 0$]; formation of the depletion region was guaranteed while the positive voltage $V_{A}=+V_{bi}$ led to a zero depletion region width. The depletion zone’s width depended mainly on two parameters; the bias voltage and the carrier density. This formula was valid for the static regime when under a fixed given bias and was considered to be insensitive to the electric field of the incident excitation.

According to Equation (1), there was a strong frequency dependency in the dielectric function of the doped GaAs bulk medium. As shown in Figure 1a, the p–n junction was bound by ideal metal layers and was excited by a transverse magnetic (TM) mode in the *xz*-plane as a point source. The amplitude of the source was small enough that the width of the depletion region was not affected by the amplitude of the source (i.e., the dynamic field did not affect the static field caused by the applied bias). To compute the charge distribution, the top/bottom metal contacts were used to assign boundary conditions for solving the Poisson’s equation from which one could extract the spatial charge distribution. Figure 2b showed the depletion region width as a function of bias and carrier density for symmetrical doping. The results in Figure 2b suggest that the maximum depletion region width could be achieved for low and moderate doping in the presence of a bias, where $V_{A}=-V_{bi}$. For the case of heavy doping, a near-zero depletion region was created, i.e., the depletion zone had negligible width (very small screening length). In Figure 2c it can be seen that the depletion region was reduced to half (i.e., maximum value of 100 nm) in the positive bias voltages. As could be expected based on the equation of the depletion zone, Figure 2c showed that the minimum voltage (i.e., zero) provided the maximum depletion zone for this positive voltage range.

To study a GaAs-based semiconductor plasmonic waveguide, equipped with the generic dielectric functions derived in the previous section, we solved Maxwell’s equations and considered the TM mode excitation for the configuration shown in Figure 2a, to obtain the relevant dispersion relation. For, w/2≤z≤d−w/2:

(2) $$\begin{array}{l} H_{y3}(\omega ,V,z)=e^{i\beta (\omega ,V,z)x}\left\{\begin{array}{l} A_{1}\cos[k_{3}(\omega ,V).(d-z)]+\\ A_{2}\sin[k_{3}(\omega ,V).(d-z)] \end{array}\right\}\\ E_{x3}(\omega ,V,z)=\frac{-ik_{3}e^{i\beta (\omega ,V,z)x}}{\omega \varepsilon_{0}\varepsilon_{3}(\omega ,V)}\left\{\begin{array}{l} A_{1}\sin[k_{3}(\omega ,V).(d-z)]-\\ A_{2}\cos[k_{3}(\omega ,V).(d-z)] \end{array}\right\}\\ E_{z3}(\omega ,V,z)=\frac{-\beta e^{i\beta (\omega ,V,z)x}}{\omega \varepsilon_{0}\varepsilon_{3}(\omega ,V)}\left\{\begin{array}{l} A_{1}\cos[k_{3}(\omega ,V).(d-z)]+\\ A_{2}\sin[k_{3}(\omega ,V).(d-z)] \end{array}\right\} \end{array}$$

for, −w/2≤z≤w/2:

(3) $$\begin{array}{l} H_{y1}(\omega ,V,z)=e^{i\beta (\omega ,V,z)x}\left\{\begin{array}{l} C_{1}\cos[k_{1}(\omega ,V).(d-z)]+\\ C_{2}\cos[k_{1}(\omega ,V).(d+z)] \end{array}\right\}\\ E_{x1}(\omega ,V,z)=\frac{-ik_{1}e^{i\beta (\omega ,V,z)x}}{\omega \varepsilon_{0}\varepsilon_{1}}\left\{\begin{array}{l} C_{1}\sin[k_{1}(\omega ,V).(d-z)]-\\ C_{2}\sin[k_{1}(\omega ,V).(d+z)] \end{array}\right\}\\ E_{z1}(\omega ,V,z)=\frac{-\beta (\omega ,V,z)e^{i\beta (\omega ,V,z)x}}{\omega \varepsilon_{0}\varepsilon_{1}}\left\{\begin{array}{l} C_{1}\cos[k_{1}(\omega ,V).(d-z)]+\\ C_{2}\cos[k_{1}(\omega ,V).(d+z)] \end{array}\right\} \end{array}$$

and for, −w/2≤z≤w/2−d:

(4) $$\begin{array}{c} H_{y2}(\omega ,V,z)=e^{i\beta (\omega ,V,z)x}\left\{\begin{array}{l} B_{1}\cos[k_{2}(\omega ,V).(d+z)]+\\ B_{2}\sin[k_{2}(\omega ,V).(d+z)] \end{array}\right\}\\ E_{x2}(\omega ,V,z)=\frac{-ik_{2}e^{i\beta (\omega ,V,z)x}}{\omega \varepsilon_{0}\varepsilon_{2}}\left\{\begin{array}{l} -B_{1}\sin[k_{2}(\omega ,V).(d+z)]+\\ B_{2}\cos[k_{2}(\omega ,V).(d+z)] \end{array}\right\}\\ E_{z2}(\omega ,V,z)=\frac{-\beta (\omega ,V,z)e^{i\beta (\omega ,V,z)x}}{\omega \varepsilon_{0}\varepsilon_{2}}\left\{\begin{array}{l} B_{1}\cos[k_{2}(\omega ,V).(d+z)]+\\ B_{2}\sin[k_{2}(\omega ,V).(d+z)] \end{array}\right\} \end{array}$$

where $k_{j}(\omega ,V)=\sqrt{\beta^{2}(\omega ,V)-k_{0}^{2}\varepsilon_{j}(\omega )}$ with *j* = 1, 2, 3. Since the tangential electric field component at the perfect electric conductor interfaces (i.e., z=±d) should be equal to zero, $A_{2}=B_{2}=0$. In addition, using the continuity of the $H_{yi}(\omega ,V,z)$ and $E_{xi}(\omega ,V,z)$ field components at z=±w/2 boundaries might result in the following SPP dispersion relation:

(5) $$\begin{array}{l} \frac{\cos[k_{1}(\omega ,V).(d-w/2)]}{\cos[k_{1}(\omega ,V).(d+w/2)]}=\\ \pm \sqrt{\frac{M_{2}(\omega ,V).\tan[k_{1}(\omega ,V).(d+w/2)]+\tan[k_{2}(\omega ,V).(d-w/2)]}{M_{2}(\omega ,V).\tan[k_{1}(\omega ,V).(d-w/2)]-\tan[k_{2}(\omega ,V).(d-w/2)]}}\times \\ \sqrt{\frac{M_{3}(\omega ,V).\tan[k_{1}(\omega ,V).(d+w/2)]+\tan[k_{3}(\omega ,V).(d-w/2)]}{M_{3}(\omega ,V).\tan[k_{1}(\omega ,V).(d-w/2)]-\tan[k_{3}(\omega ,V).(d-w/2)]}}, \end{array}$$

where $k_{j}(\omega ,V)=\sqrt{\beta^{2}(\omega ,V)-k_{0}^{2}\varepsilon_{j}(\omega )}$ with *j* = 1–3, and $M_{2,3}(\omega ,V)=k_{1}(\omega ,V)/k_{2,3}(\omega ,V)\times \varepsilon_{2,3}(\omega )/\varepsilon_{1}(\omega )$. According to Equation (5), if we insert w=2d, i.e., the entire space between the metallic plates becomes intrinsic GaAs and no electromagnetic mode can propagate inside the diode because Equation (5) has no solution. Moreover, according to Equation (5), it can be seen that, unlike the MIM waveguide structures, in the p–n junction diode, only the even plasmonic modes can be excited due to the presence of the cosine function. In this manuscript, the existence and properties of the propagating modes for the GaAs systems are discussed. Once the existence and properties of these modes are established, the excitation of these modes could be achieved using traditional techniques, such as Kretschmann configuration [54] or end-fire coupling [9]. In this regard, we expected that the excitation of the modes of the proposed layered GaAs system would be quite similar to a traditional metal-insulator-metal (MIM) system.

## 4. Results

### 4.1. Symmetric Doping Densities

**Figure 3:**
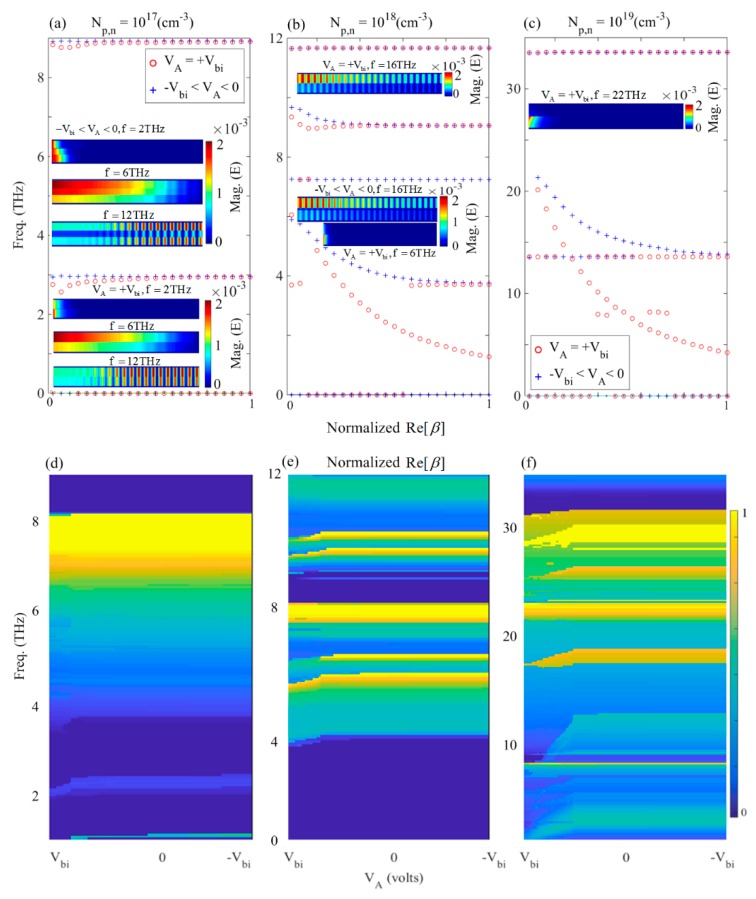
Dispersion curve peaks of the p–n junction with applied bias voltages of $V_{A}=+V_{bi}$ (circles), and $-V_{bi}<V_{A}<0$ (crosses), for carrier densities (**a**) $N_{p,n}=10^{17}({\mathrm{cm}}^{-3})$; (**b**) $N_{p,n}=10^{18}({\mathrm{cm}}^{-3})$; and (**c**) $N_{p,n}=10^{19}({\mathrm{cm}}^{-3})$ symmetric doping, respectively. The relevant two dimensional variation of the normalized dispersion curve obtained theoretically using Equation (3) for (**d**) $N_{p,n}=10^{17}({\mathrm{cm}}^{-3})$, (**e**) $N_{p,n}=10^{18}({\mathrm{cm}}^{-3})$; and (**f**) $N_{p,n}=10^{19}({\mathrm{cm}}^{-3})$ symmetric doping, respectively.

In addition to the theoretical dispersion relations given in the previous section, we carried out numerical simulations to obtain the dispersion results that are provided in Figure 3a–c. For the numerical simulation of the proposed heterostructure waveguide, a full-wave, finite-difference time-domain (FDTD) method has been used in this manuscript. A uniform discretization of the system with unit cell dimensions of 10 nm was used throughout the computational domain, as no further mesh refinement method was needed throughout the computation. The computational grid had a finite size of 60 × 1 ${\left(\mu m\right)}^{2}$ with boundary conditions corresponding to uniaxial, anisotropic, perfectly matched layers (PMLs), where 16 PMLs were used to render the absorbing boundary conditions. The computation time was set as *t* = 20,000 fs with time-step Δt = 0.87 fs, which satisfied the Cournat-Friedrichs-Lewy (CFL) stability factor condition of $\Delta t\leq 1/c\sqrt{\Delta x^{-2}+\Delta y^{-2}}$, in which c is the speed of light in free space. The waveguide was excited with a broadband dipolar point source as an oscillating electric dipole along the direction of wave propagation (*x*-axis) at $f_{0}$ = 6.5 THz, with the pulse length of 166 fs and spectral bandwidth of 11 THz. Figure 3a–c show the normalized dispersion curve peaks of the p–n junction diode obtained from the finite-difference time-domain (FDTD) simulations for the carrier densities of (a) $N_{p,n}=10^{17}({\mathrm{cm}}^{-3})$, (b) $N_{p,n}=10^{18}({\mathrm{cm}}^{-3})$, and (c) $N_{p,n}=10^{19}({\mathrm{cm}}^{-3})$ in the case of symmetrical doping, and external bias voltages of $V_{A}=+V_{bi}$ (circles), and $-V_{bi}<V_{A}<0$ (crosses), respectively. Our simulations showed that there was no difference in the dispersion curves for the negative voltages (i.e. $-V_{bi}<V_{A}<0$).

As shown in Figure 3a–c, the asymptotic frequencies of the low-doping density, such as $N_{p,n}=10^{17}({\mathrm{cm}}^{-3})$ were displayed for positive, and negative bias voltages, which corresponded to the situation where the depletion zone width for 0 and negative bias smaller than *V_bi_* did not have any notable difference. In other words, in this case, relatively small plasmon frequency intervals of *f* = 2.57 THz to 2.95 THz and *f* = 8.76 to 8.92 THz existed between the zero and non-zero depletion width, when $V_{A}=+V_{bi}$ and $-V_{bi}<V_{A}<0$, respectively. According to Figure 3b,c, for the doping densities of $N_{p,n}=10^{18}({\mathrm{cm}}^{-3})$ and $N_{p,n}=10^{19}({\mathrm{cm}}^{-3})$, the asymptotes could cover wider frequency bands, especially in the lower frequencies. This implied a wider spectral regime of propagation. For example, in the case of $N_{p,n}=10^{18}({\mathrm{cm}}^{-3})$, it was obvious that the asymptotes could cover the frequencies between *f* = 3.71 to 5.89 THz and *f* = 9.05 to 9.66 THz for $-V_{bi}<V_{A}<0$, while for $V_{A}=+V_{bi}$ a wider band between *f* = 1.27 THz to 6.06 THz and *f* = 8.97 THz to 9.34 THz was covered, respectively. 

Non-plasmonic modes emerged beyond *f* ≈ 43 THz on the left side of the light line, due to the cut-off frequencies of the metallic waveguide-like behavior of the diode. Therefore, we concentrated on the lower frequencies to investigate the depletion-zone-dependent effects, under the negative and positive bias voltages.

Figure 3c shows that for $N_{p,n}=10^{19}({\mathrm{cm}}^{-3})$, the asymptotes emerged at higher frequencies due to the greater plasmon frequency that resulted from the higher doping values. The asymptotes covered a wider frequency band between *f* = 4.21 THz to 20.13 THz for $V_{A}=+V_{bi}$, in comparison to *f* = 13.6 THz to 21.33 THz band for $-V_{bi}<V_{A}<0$. This feature was useful for the purpose of filtering in the nano-photonics integrated circuits, at the THz regime. For the asymptotic case with w=0 occurring under $V_{A}=+V_{bi}$, theoretically two conditions can exist: (1) $\tan(k_{1}d)=0$, and (2) a transcendental equation of the $\left(k_{3}/\varepsilon_{3})\times \tan(k_{3}d\right)$ + $(k_{2}/\varepsilon_{2})\times \tan(k_{2}d)=0$ condition. Since under this voltage there is no depletion region, the first condition might not be satisfied and only the second condition can exist at some frequencies. To theoretically study the p–n junction waveguide, we plotted the solution of Equation (3) versus the bias voltage. Figure 3d–f illustrate the normalized dispersion curve using an interior, subspace conjugate gradient method [55] obtained for (d) $N_{p,n}=10^{17}({\mathrm{cm}}^{-3})$, (e) $N_{p,n}=10^{18}({\mathrm{cm}}^{-3})$, and (f) $N_{p,n}=10^{19}({\mathrm{cm}}^{-3})$, respectively. It can be seen that as mentioned earlier, using the simulation results (Figure 3a–c), the dispersion curve was constant for whole voltage region of w≠0 i.e., $-V_{bi}<V_{A}<0$, and was different for the case of w=0, i.e., for $V_{A}=+V_{bi}$. Although there were some frequency deviations and ripples in the dispersion curves, for the high-doping values (Figure 3e,f), the results supported a notable wave-guiding trend in the p–n junction. Therefore, based on the simulation and theoretical results shown in Figure 3a–f, it could be stated that unlike the MIM waveguide structures wherein the thickness of the insulator layer determined the propagation wavelength of the wave, for the diode waveguide; existence or lack of the depletion zone could change the frequency of the propagating plasmon wave. The results in Figure 3 suggest that increasing the doping density results in the blue-shift of the asymptotic plasmonic frequencies. The insets of Figure 3a–c show the distribution of absolute value of the $E_{x}$ component of the electric field, inside the waveguide. It can be seen for example that in the case of $N_{p,n}=10^{17}({\mathrm{cm}}^{-3})$, although the dispersion curve did not represent an asymptotic frequency at *f* = 2 THz, the imaginary part of the individual dielectric functions were so high (see Figure 1a–d) that the wave could not propagate inside the waveguide and got rapidly damped.

For the frequency bands between the asymptotic frequencies and the first traditional cut-off frequency of the metallic waveguide, i.e., *f* = 6 THz and 12 THz, respectively, the wave could propagate much more easily, due to the lower propagation loss of the doped mediums and near-zero-epsilon conditions (see Figure 1a–d). Similarly, the same situation governed the wave propagation for $N_{p,n}=10^{18}({\mathrm{cm}}^{-3})$; at *f* = 6 THz and 16 THz; and $N_{p,n}=10^{19}({\mathrm{cm}}^{-3})$ at *f* = 22 THz, in Figure 3b,c, respectively.

**Figure 4:**
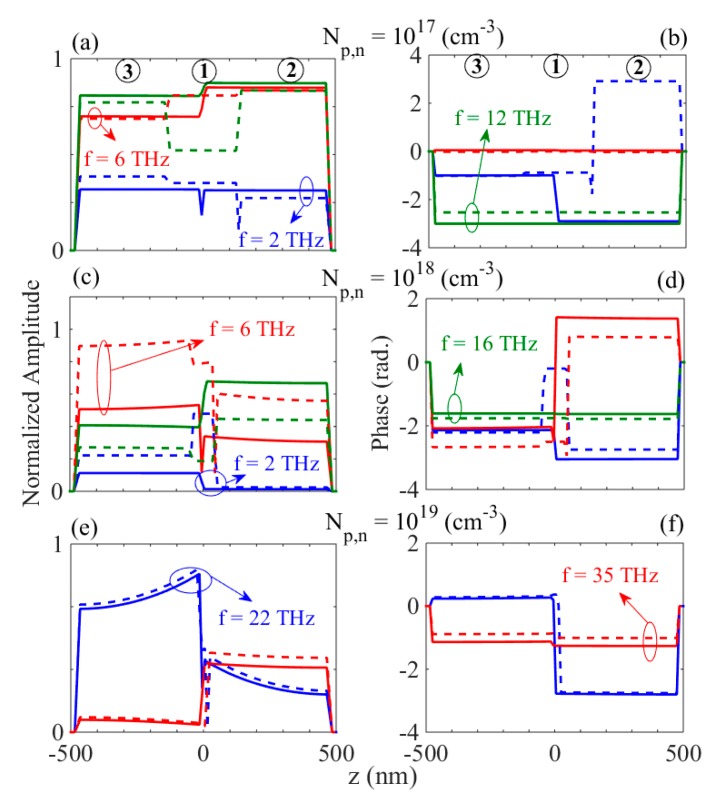
The normalized electric field amplitude and phase variation inside the p–n junction waveguide for the individual $V_{A}=+V_{bi}$ (solid-curves), and $V_{A}=-V_{bi}$ (dashed-curves) (**a**,**b**) $N_{p,n}=10^{17}({\mathrm{cm}}^{-3})$ at *f* = 2 THz (blue-line), 6 THz (red-line), and 12THz (green-line); (**c**,**d**) $N_{p,n}=10^{18}({\mathrm{cm}}^{-3})$ at *f* = 2 THz (blue-line), 6 THz (red-line), and 16 THz (green-line); (**e**,**f**) $N_{p,n}=10^{19}({\mathrm{cm}}^{-3})$ at *f* = 22 THz (blue-line), 35 THz (red-line), respectively. The regions (1), (2), and (3) correspond to the depletion region, n-doped region, and p-doped region, respectively (see Figure 2a).

Based on the insets of Figure 3a–c, it was observed that, due to the different dispersion properties of the p- and n-doped regions at a certain frequency, the electric field distribution in each of the doped regions were different, as expected. Therefore, the electric field at a given frequency of the excitation experienced various phase differences in each of the regions. Figure 4a–f show the normalized amplitude and the relevant phase variations of the $E_{x}$ component of the electric field along the *z*-direction for $V_{A}=+V_{bi}$ (solid-curves) and $-V_{bi}<V_{A}<0$ (dashed-curves) for different doping levels at various frequencies. 

It should be noted that Figure 1a–d depict the optical properties of the bulk GaAs medium without any surface or boundary effects, unlike the hetereostructure investigated in this work. Figure 1 serves as a basis for the calculations undertaken for the finite heterostructure. The fields plotted in Figure 4a–f are related to the waveguide structure, where the boundary conditions and surface effects have been considered. In Figure 4a,b for $N_{p,n}=10^{17}({\mathrm{cm}}^{-3})$ it can be seen that at *f* = 2 THz, although the amplitudes are approximately equal, the phase difference of the electric fields in media 3 and 2 for the positive and negative biases are between π/2 (rad.) and π (rad.). However, for the positive bias voltage at *f* = 6 THz and 12 THz, a zero phase difference and approximately equal amplitudes were obtained, consequently preventing destructive interference effects, and the propagating solutions were damped at larger distances. Similarly, for $N_{p,n}=10^{18}({\mathrm{cm}}^{-3})$, using Figure 4c,d the phase difference of the propagating $E_{x}$ component in media 1, 2, and 3 at *f* = 16 THz was equal to zero, whereas, the amplitudes were slightly different especially in the case of the negative voltage. It seemed that this issue arose due to the smaller difference of the imaginary parts of the semiconductor media in the case of $N_{p,n}=10^{17}({\mathrm{cm}}^{-3})$ and $N_{p,n}=10^{18}({\mathrm{cm}}^{-3})$, and at frequencies greater than $\omega_{TO}$. On the other hand, this behavior was not observed for $N_{p,n}=10^{19}({\mathrm{cm}}^{-3})$ (see Figure 4e,f).

### 4.2. Asymmetric Doping Densities

In this section, the optical properties of the waveguide under asymmetric carrier concentration are discussed. Here, we showed that the decoherence effects due to the difference in the effective masses of electrons and holes in the n- and p-doped regions were further enhanced due to asymmetrical doping. In addition to the differences between the plasmon frequencies, since the electron and hole mobilities in the p- and n-doped regions were considerably different, the carrier relaxation time and hence the collision rate of these regions also differed. These effects eventually led to different dielectric functions of the doped mediums, which in turn, disturbed the propagated field inside the p–n junction waveguide. Thus, in order to avoid such effects, the equal dielectric function condition of the p- and n-GaAs based on Equation (1); i.e., Re[εp−GaAs(ω)]=Re[εn−GaAs(ω)] needed to be achieved:

(6) $$\left(\begin{array}{c} N_{p}\\ N_{n} \end{array}\right)=\left(\begin{array}{c} N_{n}\\ N_{p} \end{array}\right)\times \left(\begin{array}{c} m_{p}^{*}/m_{n}^{*}\\ m_{n}^{*}/m_{p}^{*} \end{array}\right)\times \left\{\begin{array}{c} \left(\frac{\omega^{2}+\frac{e^{2}}{4\pi^{2}\cdot \mu_{p}^{2}\cdot m_{p}^{*}}}{\omega^{2}+\frac{e^{2}}{4\pi^{2}\cdot \mu_{n}^{2}\cdot m_{n}^{*}}}\right)\\ \left(\frac{\omega^{2}+\frac{e^{2}}{4\pi^{2}\cdot \mu_{n}^{2}\cdot m_{n}^{*}}}{\omega^{2}+\frac{e^{2}}{4\pi^{2}\cdot \mu_{p}^{2}\cdot m_{p}^{*}}}\right) \end{array}\right\}$$

Since we were interested in small amplitudes of the excitation field that did not change the width of the depletion region, we concentrated on the carrier densities in the static regime:

(7) $$\left(\begin{array}{c} N_{p}\\ N_{n} \end{array}\right)=\left(\begin{array}{c} N_{n}\\ N_{p} \end{array}\right)\cdot \left(\begin{array}{c} m_{n}^{*}/m_{p}^{*}\\ m_{p}^{*}/m_{n}^{*} \end{array}\right)\cdot {\left(\begin{array}{c} \mu_{n}/\mu_{p}\\ \mu_{p}/\mu_{n} \end{array}\right)}^{2}$$

In Equation (7), the carrier density relation, which resulted in the same relative permittivity in the static situation, strongly depended on the ratio of the effective masses and square of ratios of the electron and hole mobilities, respectively. To achieve an equal dielectric function in both regions, the n-region doping values of $N_{n}=10^{17}({\mathrm{cm}}^{-3})$, $N_{n}=10^{18}({\mathrm{cm}}^{-3})$, and $N_{n}=10^{19}({\mathrm{cm}}^{-3})$ should correspond to a p-region doping ratio of $N_{p}=7.5\times 10^{18}({\mathrm{cm}}^{-3})$, $N_{p}=7.5\times 10^{19}({\mathrm{cm}}^{-3})$, and $N_{p}=7.5\times 10^{20}({\mathrm{cm}}^{-3})$, for weak, moderate, and heavy doping, respectively.

**Figure 5:**
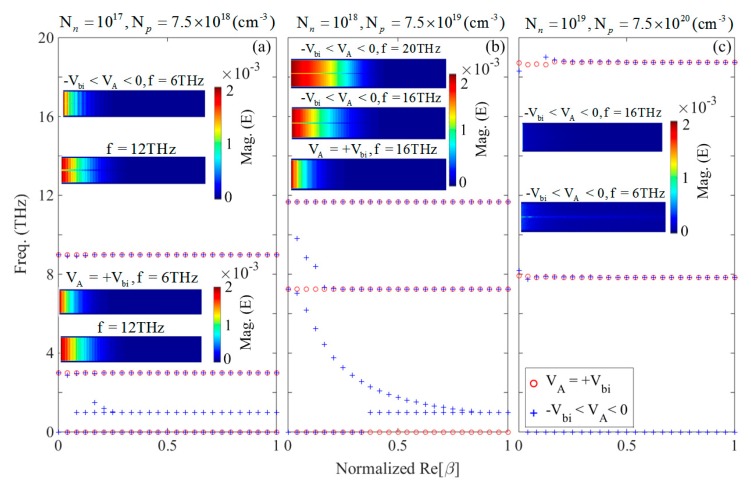
Dispersion curve peaks of the p–n junction with applied bias voltages of $V_{A}=+V_{bi}$ (circles), and $-V_{bi}<V_{A}<0$ (crosses) achieved from the simulations, for carrier densities (**a**) $N_{n}=10^{17}({\mathrm{cm}}^{-3})$ and $N_{p}=7.5\times 10^{18}({\mathrm{cm}}^{-3})$; (**b**) $N_{n}=10^{18}({\mathrm{cm}}^{-3})$ and $N_{p}=7.5\times 10^{19}({\mathrm{cm}}^{-3})$; and (**c**) $N_{n}=10^{19}({\mathrm{cm}}^{-3})$ and $N_{p}=7.5\times 10^{20}({\mathrm{cm}}^{-3})$ doping densities, respectively. The insets show the amplitude of $E_{x}$ component at *f* = 6 THz and 12 THz for $N_{n}=10^{17}({\mathrm{cm}}^{-3})$ and $N_{p}=7.5\times 10^{18}({\mathrm{cm}}^{-3})$, at *f* = 16 THz and 20 THz for $N_{n}=10^{18}({\mathrm{cm}}^{-3})$ and $N_{p}=7.5\times 10^{19}({\mathrm{cm}}^{-3})$, and at *f* = 6 THz and 16 THz for $N_{n}=10^{19}({\mathrm{cm}}^{-3})$ and $N_{p}=7.5\times 10^{20}({\mathrm{cm}}^{-3})$, respectively.

Figure 5a–c demonstrate the dispersion curve peaks for the p–n junction waveguide with $N_{n}=10^{17}({\mathrm{cm}}^{-3})$ and $N_{p}=7.5\times 10^{18}({\mathrm{cm}}^{-3})$ (Figure 5a); $N_{n}=10^{18}({\mathrm{cm}}^{-3})$ and $N_{p}=7.5\times 10^{19}({\mathrm{cm}}^{-3})$ (Figure 5b); $N_{n}=10^{19}({\mathrm{cm}}^{-3})$ and $N_{p}=7.5\times 10^{20}({\mathrm{cm}}^{-3})$ (Figure 5c) doping densities, for $V_{A}=+V_{bi}$ (circles) and $-V_{bi}<V_{A}<0$ (crosses), respectively. According to Figure 5a–c, for the case of the positive voltage, the asymptotic frequencies were negligibly blue-shifted in comparison to the symmetric doping case. For example, for $N_{n}=10^{17}({\mathrm{cm}}^{-3})$ and $N_{p}=7.5\times 10^{18}({\mathrm{cm}}^{-3})$, we had the asymptotes of *f* = 2.98 THz and 8.97 THz which occurred at *f* = 2.96 THz and 8.91 THz for $N_{p,n}=10^{17}({\mathrm{cm}}^{-3})$, and we also obtained *f* = 7.27 THz and 11.69 THz for $N_{n}=10^{18}({\mathrm{cm}}^{-3})$ and $N_{p}=7.5\times 10^{19}({\mathrm{cm}}^{-3})$, while we saw asymptotes at *f* = 7.24 THz and 11.65 THz for $N_{p,n}=10^{18}({\mathrm{cm}}^{-3})$ symmetric doping densities, respectively.

Furthermore, based on Figure 5a–c it is obvious that in the case of applied negative bias, another asymptotic frequency at *f* = 1 THz originated for $N_{n}=10^{17}({\mathrm{cm}}^{-3})$ and $N_{p}=7.5\times 10^{18}({\mathrm{cm}}^{-3})$, and also for $N_{n}=10^{18}({\mathrm{cm}}^{-3})$ and $N_{p}=7.5\times 10^{19}({\mathrm{cm}}^{-3})$. Unlike the symmetric doping densities, for the asymmetric case, the positive voltage could not support a substantial wide region of plasmonic asymptotic frequencies. For $N_{n}=10^{18}({\mathrm{cm}}^{-3})$ and $N_{p}=7.5\times 10^{19}({\mathrm{cm}}^{-3})$, with negative voltages we could achieve an ultra-wide asymptotic frequency band of 8.82 THz between *f* = 1 THz and 9.82 THz. Although, in the asymmetric doping densities the electric field inside the *pn*-junction waveguide is uniform and in-phase along the *z*-axis in both the *p*- and *n*-doped medium, the insets of the Figure 5a–c reveal that the electromagnetic field at a certain frequency cannot propagate as easily as it does in the case of the asymmetric doping at the relevant frequency.

## 5. Conclusions

In this work, we derived a dispersion relation for the p–n heterojunction and applied the resulting relations to a GaAs-based p–n junction using the material constants and band parameters from the existing literature. With the use of the dispersion curves and by carrying out numerical simulations, we showed that better tunability could be achieved at frequencies between the TO phonon resonance frequency and the first cut-off frequency of GaAs-filled metallic waveguide. We theoretically and numerically demonstrated that the p–n junction waveguide, unlike the MIM waveguides, supported both plasmonic asymptotic and cut-off frequencies of the traditional waveguide, in the THz regime. We also showed that highly asymmetric doping levels might cause phase shifts of the propagating plasmon waves in the n- and p-doped regions that lead to the loss of coherence of the propagating waves. Our findings show the way for doped p–n junctions or similar heterostructures that can be tailored for a variety of tunable optoelectronic applications. Such features of p–n junction waveguides hold promise for low-loss, wide bandwidth optoelectronic applications in the THz spectrum, and can act as efficient interfaces between ICs and optics.

## Figures and Tables

**Table 1:** The optical parameters of the GaAs medium used in Equation (1).

$\varepsilon_{DC,GaAs}$	$\varepsilon_{\infty ,GaAs}$	$\omega_{TO}(\mathrm{THz})$	$\omega_{LO}(\mathrm{THz})$	Γ(THz)
12.9	10.9	8	8.5	0.055
